# 

**Published:** 1907-02

**Authors:** 


### BOOKS RECEIVED.

International Clinics. A quarterly of Illustrated Clinical Lectures and especially prepared articles on Treatment, Medicine, Surgery, Neurology, Pediatrics, Obstetrics, Gynecology, Orthopedics, Pathology, Dermatology, Ophthalmology, Otology, Rhinology, Laryngology, Hygiene and other topics of interest to students and practitioners. By leading members of the medical profession throughout the world. Edited by A. O. J. Kelly, A.M., M.D. Volume IV. Sixteenth series. 1906. Philadelphia and London: J. B. Lippincott Co. (Cloth, $2.00).

---

Woman in Girlhood, Wifehood and Motherhood. Her responsibilities and her duties at all periods of life and a guide in the maintenance of her own health and that of her children. By M. Solis-Cohen, A.B., M.D., Instructor in Physical Diagnosis, University of Pennsylvania. Profusely illustrated with color-plates, scientific drawings and half-tone engravings and manikin chart printed in colors. Octavo, pp. 500. The John C. Winston Company, Philadelphia. (Price, $2.00).

---

The Practice of Obstetrics. By Eminent Authorities. Edited by Reuben Peterson, A.B., M.D., Professor of Obstetrics and Diseases of Women in the University of Michigan, Department of Medicine and Surgery, Ann Arbor, Mich. Large octavo, about 1087 pages, with 523 engravings and 30 full-page plates in colors and monochrome. Lea Brothers & Co., Philadelphia and New York, 1907. (Cloth, $6.00; leather, $7.00; half morocco, $8.00, net prices).

---

The Practical Medicine Series of Year Books. Ten volumes. Isued under the general editorial charge of Gustavus P. Head, M.D., Professor of Laryngology and Rhinology in the Chicago Post-Graduate Medical School. Vol. IX. Anatomy, Physiology, Pathology, Dictionary. Edited by W. A. Evans, Adolph Gehrmann, William Healy. Series 1906. Chicago: The Year Book Publishers. (Price, $1.25; entire series, $10.00).

The Practical Medicine Series of Year Books. Ten Volumes. Issued under the general editorial charge of Gustavus P. Head, M.D., Professor of Laryngology and Rhinology in the Chicago Post-Graduate Medical School. Vol. X. Skin and Venereal Diseases, Nervous and Mental Diseases. Edited by W. L. Baum, Hugh T. Patrick, William Healy. Series 1906. Chicago: The Year Book Publishers. (Price, $1.25; entire series, $10.00).

---

A Treatise on Orthopedic Surgery. By Royal Whitman, M.D., Instructor in Orthopedic Surgery in the College of Physicians and Surgeons, New York; Chief of Orthopedic Department in Vanderbilt Clinic, New York. Third edition, revised and enlarged. Octavo, 900 pages, with 554 illustrations, mostly original. Lea Brothers & Co., Philadelphia and New York, 1907. (Cloth, $5.50 net).

---

Starr on Nervous Diseases. Organic and Functional Nervous Diseases. By M. Allen Starr, M.D., Ph.D., LL.D., Professor of Neurology in the College of Physicians and Surgeons, New York. Second edition, revised. Octavo, 824 pages, with 282 engravings and 26 full-page plates. Lea Brothers & Co., Philadelphia and New York, 1907. (Cloth, $6.00, net; leather, $7.00, net).

---

Lea's Series of Pocket Textbooks. Diseases of Children. By George M. Tuttle, M.D., Attending Physician to St. Luke's Hospital, St. Louis, Mo. 2d edition, revised. 12mo, 392 pages, with 5 plates. Edited by Bern. B. Gallaudet, M.D. Lea Brothers & Co., Philadelphia and New York, 1907. (Cloth, $1.50; flexible leather, $2.00 net prices).

---

Syllabus of Lectures on Human Embryology. With a Glossary of Embryological Terms. By Walter Porter Wanton, M.D., Professor of Clinical Gynecology and Professor adjunct of Obstetrics in the Detroit College of Medicine. Third edition. Illustrated. 12mo., pp. 136. Philadelphia: F. A. Davis Co. 1906.

---

Plaster of Paris and How to Use It. By Martin W. Ware, M.D., Adjunct Attending Surgeon, Mount Sinai Hospital; Surgeon to the Good Samaritan Dispensary; Instructor in Surgery, N. Y. Post-Graduate Medical School. 12mo; 72 illustrations, about 100 pages. New York: Surgery Publishing Co. (Cloth, $1.00).

---

Practical Dietetics, with reference to Diet in Disease. By Alida Frances Pattee, Late Instructor in Dietetics, Bellevue Training School for Nurses, New York. Fourth edition, 12mo, cloth. 300 pages. New York: A. F. Pattee, Publisher, 52 West 39th Street. (Price, $1.00 net).

---

Tumors of the Cerebrum. Their Focal Diagnosis and Surgical Treatment. By Charles K. Mills, Charles H. Frazier, William G. Spiller, George E. de Schweinitz, and Theodore H. Weisenburg. Small 8 vo., pp. 35. Philadelphia: Edward Bennock, 3609 Woodland Avenue. 1906.

---

Woman. A Treatise on the Normal and Pathological Emotions of Feminine Love. By Bernard S. Talmey, M.D., Gynecologist to the Metropolitan Hospital and Dispensary, New York. 12mo., pp. 300. Illustrated. New York: Stanley Press Co. (Price, $3.00).

Conservative Gynecology and Electro-Therapeutics. By G. Betton Massey, M.D., Attending Surgeon to the American Oncologic Hospital, Phiadelphia. Fifth edition. Illustrated. Octavo, pp. 467. Philadelphia: F. A. Davis Co. 1906.

---

Diseases of the Stomach and Intestines. By Boardman Reed, M.D., Late Physician in Chief to the Samaritan Hospital, Philadelphia. Second edition. Illustrated. Octavo, pp. 1021. New York: E. B. Treat & Co. 1907. (Price, $5.00).

---

The Hygiene of Pregnancy. By Walter B. Jennings, M.D., Attending Physician to St. Mary's Free Hospital for Children. New York: Medical Review of Reviews. (Price, 25 cents).

---

Transactions of the American Surgical Association. Vol. 24. Edited by Richard H. Harte, M.D., Recorder of the Association. Philadelphia: William J. Dornan, Printer.

---

The Harvey Lectures, delivered under the auspices of the Harvey Society of New York. 1905-1906. Philadelphia and London: J. B. Lippincott Co.

---

Transactions of the Maine Medical Association. Vol. XV. Part III. Walter E. Tobie, M.D., Secretary.

